# SARS-CoV-2 natural infection in animals: a systematic review of studies and case reports and series

**DOI:** 10.1080/01652176.2021.1970280

**Published:** 2021-09-02

**Authors:** D. Katterine Bonilla-Aldana, Alejandra García-Barco, S. Daniela Jimenez-Diaz, Jorge Luis Bonilla-Aldana, Maria C. Cardona-Trujillo, Fausto Muñoz-Lara, Lysien I. Zambrano, Luis A. Salas-Matta, Alfonso J. Rodriguez-Morales

**Affiliations:** a Semillero de Investigación en Zoonosis (SIZOO), Grupo de Investigación GISCA, Fundación Universitaria Autónoma de las Américas, Pereira, Risaralda, Colombia; b Grupo Colaborativo de Investigación en Enfermedades Transmitidas por vectores, Zoonóticas y tropicales de Risaralda, Pereira, Risaralda, Colombia; c School of Veterinary Medicine and Zootechnics, Universidad de la Amazonia, Florencia, Caquetá, Colombia; d Department of Internal Medicine, Faculty of Medical Sciences, Universidad Nacional Autónoma de Honduras, Tegucigalpa, Honduras; e Department of Internal Medicine, Hospital Escuela, Tegucigalpa, Honduras; f Unit of Scientific Research, School of Medicine, Faculty of Medical Sciences, Universidad Nacional Autónoma de Honduras (UNAH), Tegucigalpa, Honduras; g Faculty of Health Sciences, Universidad Científica del Sur, Lima, Perú; h Grupo de Investigación Biomedicina, Faculty of Medicine, Fundación Universitaria Autónoma de las Américas, Pereira, Risaralda, Colombia; i School of Medicine, Universidad Privada Franz Tamayo (UNIFRANZ), Cochabamba, Bolivia

**Keywords:** SARS-CoV-2, COVID-19, prevalence, animals, zoonotic, transmission

## Abstract

COVID-19 pandemic is essentially a zoonotic disease. In this context, early in 2020, transmission from humans to certain animals began reporting; the number of studies has grown since. To estimate the pooled prevalence of SARS-CoV-2 natural infection in animals and to determine differences in prevalence between countries, years, animal types and diagnostic methods (RT-PCR or serological tests). A systematic literature review with meta-analysis using eight databases. Observational studies were included but analyzed separately. We performed a random-effects model meta-analysis to calculate the pooled prevalence and 95% confidence interval (95% CI) for prevalence studies and case series. After the screening, 65 reports were selected for full-text assessment and included for qualitative and quantitative analyses. A total of 24 reports assessed SARS-CoV-2 infection by RT-PCR, combining a total of 321,785 animals, yielding a pooled prevalence of 12.3% (95% CI 11.6%–13.0%). Also, a total of 17 studies additionally assessed serological response against SARS-CoV-2, including nine by ELISA, four by PRTN, one by MIA, one by immunochromatography (rest, two studies, the method was not specified), combining a total of 5319 animals, yielding a pooled prevalence of 29.4% (95% CI 22.9%–35.9%). A considerable proportion of animals resulted infected by SARS-CoV-2, ranking minks among the highest value, followed by dogs and cats. Further studies in other animals are required to define the extent and importance of natural infection due to SARS-CoV-2. These findings have multiple implications for public human and animal health. One Health approach in this context is critical for prevention and control.

## 1. Introduction

Since the course of the pandemic of Coronavirus Disease 2019 (COVID-19), caused by the Severe Acute Respiratory Syndrome coronavirus 2 (SARS-CoV-2), there has been an interest in understanding its relationship with animal hosts (Bonilla-Aldana, Villamil-Gómez, et al. 2020), as part of its origin, but also in terms of the risk of infection from human sources (Rodriguez-Morales, Bonilla-Aldana et al. 2020; Tiwari et al. 2020; Sharun, Dhama, et al. 2021). Regardless of how clear is the first, and yet some doubts about the primary, and especially the intermediate hosts of the SARS-CoV-2 (Ahmad et al. 2020), more information has become available about the natural infection by different animals that are in close contact with humans in different scenarios, especially domestic (pet or companion animals), farming, zoos and even also in wild nature (Bonilla-Aldana, Dhama, et al. 2020; Bonilla-Aldana, Holguin-Rivera, et al. 2020; Bonilla-Aldana, Jimenez-Diaz, et al. 2020; Bonilla-Aldana et al. 2021).

During years 2020 and half of 2021, relevant case reports, case series and prevalence studies have assessed the natural infection due to SARS-CoV-2 in different animals, particularly felines, canines and Mustelidae; looking infection especially as a consequence of contact with human beings with COVID-19 (Li 2020; Rodriguez-Morales, Dhama et al. 2020; Salajegheh Tazerji et al. 2020 ). As suspected, the SARS-CoV-2 can jump or spill over into new animal species amid the current pandemic (Dhama et al. 2020; Sharun, Tiwari, et al. 2021). Moreover, many exposed animals have been infected with the SARS-CoV-2 from humans, resulting in disease and death (Halfmann et al. 2020; Izes et al. 2020; Stout et al. 2020; Hosie et al. 2021).

So far, after one and a half years after the beginning of the threat of the SARS-CoV-2/COVID-19, some of the questions remaining are how susceptible are, which is the proportion of different animals that may be infected by this emerging coronavirus, which proportion of animal species are susceptible and which proportion of animals in a group is infected (Colitti et al. 2021). According to the study, the observed range of the last is highly variable, from 0% to 100% according to the generated evidence. Then, a systematic review with meta-analysis may help understand risk and specifically to know which is the global relative frequency of natural infection due to SARS-CoV-2 in animals, mainly domestic, from farms and zoos. Unfortunately, no other systemic reviews or meta-analyses have been published on this topic to the best of our knowledge.

The objectives of this systematic review were 1) to estimate the pooled prevalence of SARS-CoV-2 natural infection in animals based on available reports and observational studies and 2) to determine differences in the prevalence between countries, years, animal types and diagnostic methods (RT-PCR or serological tests).

## 2. Methods

### 2.1. Protocol and registration

Our protocol followed the recommendations established by the Preferred Reporting Items for Systematic Reviews and Meta-Analyses (PRISMA) statement (Moher et al. 2009).

### 2.2. Eligibility criteria

We included published peer-reviewed articles that reported cases, case series, and prevalence studies with assessment of natural infection due to SARS-CoV-2 by RT-PCR and serological tests such as Enzyme-Linked ImmunoSorbent Assay (ELISA) and Plaque Reduction Neutralization Test (PRNT). Article language limit was not set, and we included publications from Jan 1, 2020, until June 1, 2021. Reviews, opinion articles, and letters not offering original data were excluded as well as studies reporting cases with incomplete information. Reports from the World Animal Health Organization (OIE) were included. If a case was later reported in a publication, it was considered duplication, and the first was removed from the inclusion in this review.

### 2.3. Information sources and search strategy

We conducted a systematic review using Medline/PubMed, Scopus, Web of Science, SciELO, LILACS, Redalyc, ScienceDirect and Google Scholar. The search terms used included: ‘coronavirus’, ‘severe acute respiratory syndrome’, ‘severe acute respiratory syndrome 2’, ‘SARS’, ‘SARS-CoV’, ‘SARS-CoV-2’, ‘animal’, ‘natural infection’ and ‘zoonotic’, with multiple combinations between them. The searches ended by June 6, 2021. Two different researchers independently evaluated the search results.

### 2.4. Study selection

**Figure 1.**
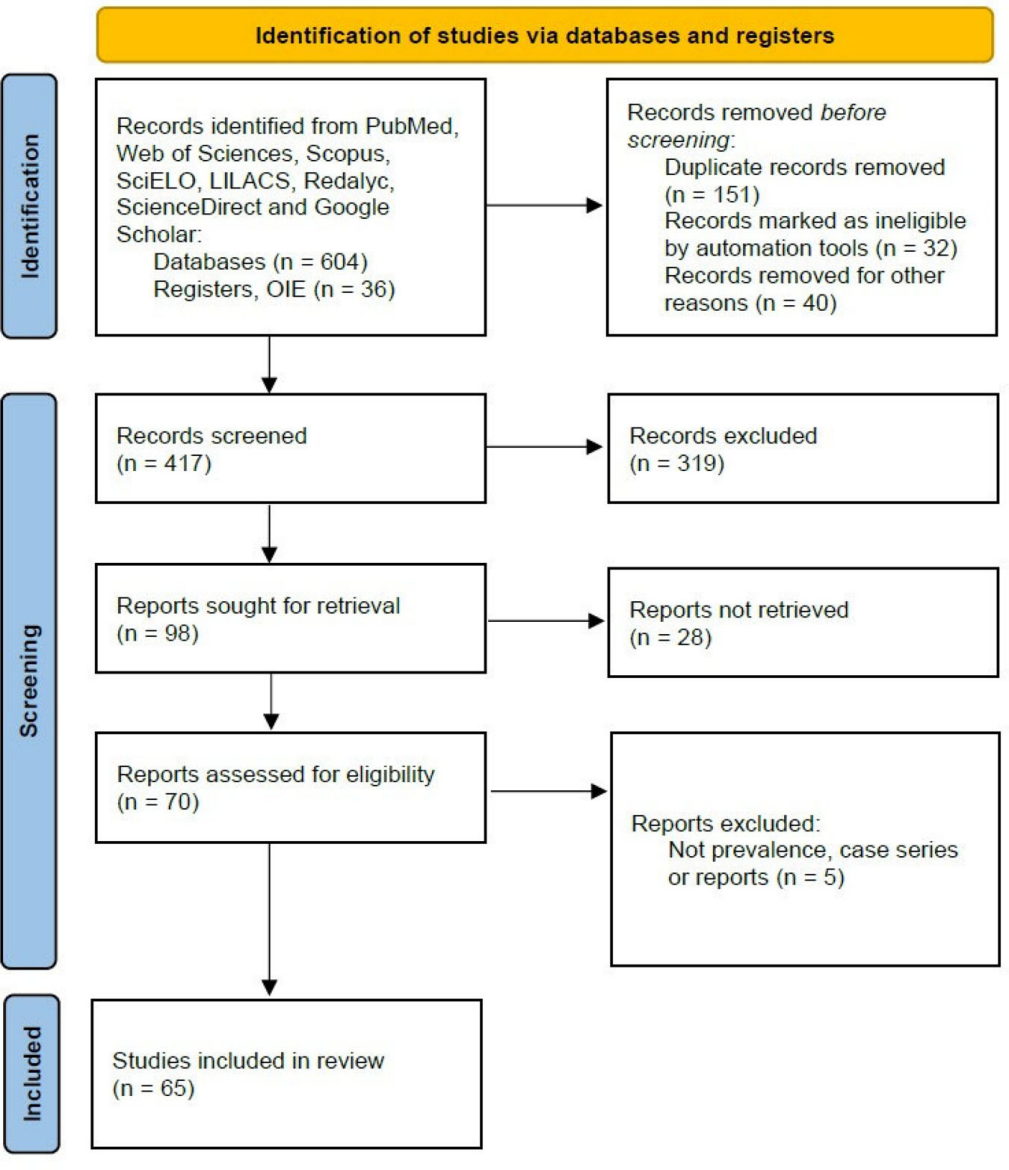
Study selection and characteristics, based on the PRISMA 2020 Standard for Systematic Reviews.

Initial search strategy results were screened by title and abstract. The full texts of relevant articles were examined for inclusion and exclusion criteria (Figure 1). When an article reported the same animal's information, reports were combined to obtain complete data and counted as a single case. Observational studies that reported the proportion of infected animals using different diagnostic methods were included for quantitative synthesis (meta-analysis).

### 2.5. Data collection process and data items

Data extraction forms, including information on the type of publication, publishing institution, country, year, date of publication, number of reported cases, animal species, samples, type of animal (domestic/pet, farm, zoo, wild), and diagnostic method, were filled independently by two researchers. A fifth investigator checked the article list and data extractions to guarantee duplicate articles or duplicate information and resolved discrepancies in study inclusion.

### 2.6. Assessment of methodological quality and risk of bias

We used the critical appraisal tool of the Quality Appraisal of Case Series Studies Checklist of the IHE to assess the quality of cross-sectional studies (AXIS) (IHE 2014; Downes et al. 2016). Publication bias was assessed using a funnel plot. A random-effects model was used to calculate the pooled prevalence, and 95% CI gave variable degrees of data heterogeneity and the inherent heterogeneity in any systematic review of studies from the published literature. Egger's and Kendall tests for publication bias was also performed.

### 2.7. Statistical approach

Unit discordance for variables was resolved by converting all units to a standard measurement for each variable. Then, percentages and means ± standard deviation (SDs) were calculated to describe the distributions of categorical and continuous variables, respectively. Since individual information was not available for all patients, we report weighted means and SDs. The baseline data were analyzed using the Stata version 14.0, licensed for Universidad Tecnológica de Pereira in Colombia.

The meta-analyses were performed using Stata, and the software OpenMeta[Analyst, Providence, Rhode Island, USA] (Wallace et al. 2012), JASP (Amsterdam, the Netherlands, Version 0.12.2)®, and Comprehensive Meta-Analysis ve.3.3® (Englewood, New Jersey, USA) licensed for Universidad Tecnológica de Pereira. Pooled prevalences and their 95% confidence intervals (95% CIs) were used to summarize the weighted effect size for each study grouping variable using a binary random-effects model (which takes into consideration sample sizes of individual studies) was applied (DerSimonian-Laird procedure) (Kontopantelis and Reeves 2012; Viechtbauer 2010).

Measures of heterogeneity, including Cochran's Q statistic, I^2^ index, and tau-squared test, were estimated and reported. We performed subgroup analyses by countries, animals and years and meta-analyses for each interest variable. Publication bias was assessed using a funnel plot.

A supplemental table with the main characteristics of included studies is available upon request.

## 3. Results

### 3.1. Study selection and characteristics

A total of 604 articles (plus 36 reports from OIE) were retrieved using the search strategy. After screening by abstract and title, 70 articles were selected for full-text assessment. Of these, five were excluded due to the lack of information on laboratory diagnosis, and 65 were finally included for the final qualitative synthesis and meta-analysis (Figure 1).

### 3.2. Molecular findings from prevalence studies

**Figure 2.**
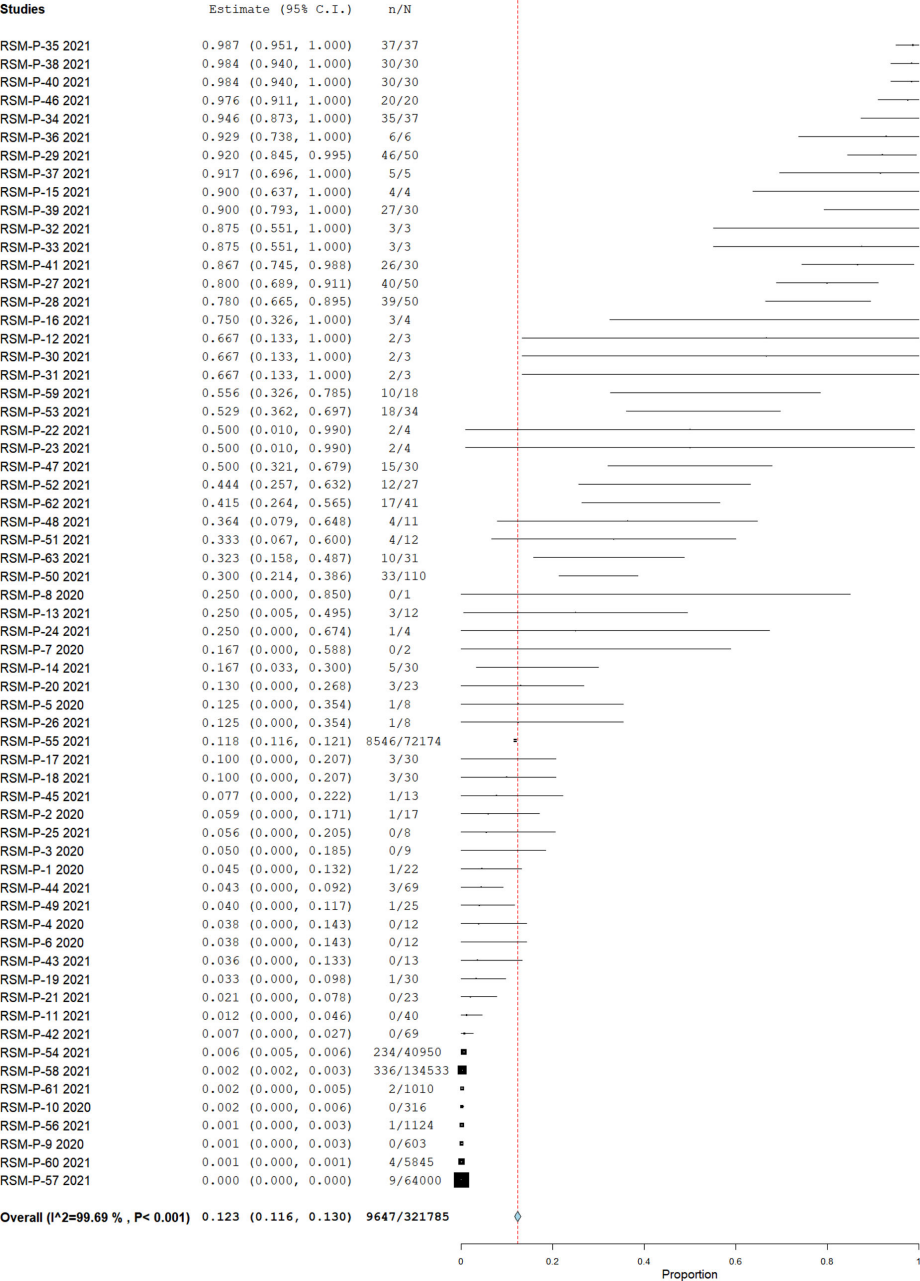
Pool prevalence of SARS-CoV-2 among animals assessed by RT-PCR from prevalence studies.

From those studies with ten or more animals, considered prevalence studies, we found 32 publications. Of them, 24 (75%) assessed SARS-CoV-2 infection by RT-PCR, combining a total of 321,785, being 9647 positives, yielding a pooled prevalence of 12.3% (95% CI 11.6%–13.0%) (Q = 20,168.3; I^2^=99.693; τ^2^<0.001; *p* < 0.001) (Figure 2); these were assessed by nasal swab sampling in 38.8% of them, 21.3% throat, 16.3% rectal, 13.8% fecal, and 10% oral. Nine of the publications reported clinical findings, combining a total of 134,611 animals (41.83%). Regarding the origin, these were 99.5% from farms, 0.42% pets/domestic, and 0.01% wild.

**Figure 3.**
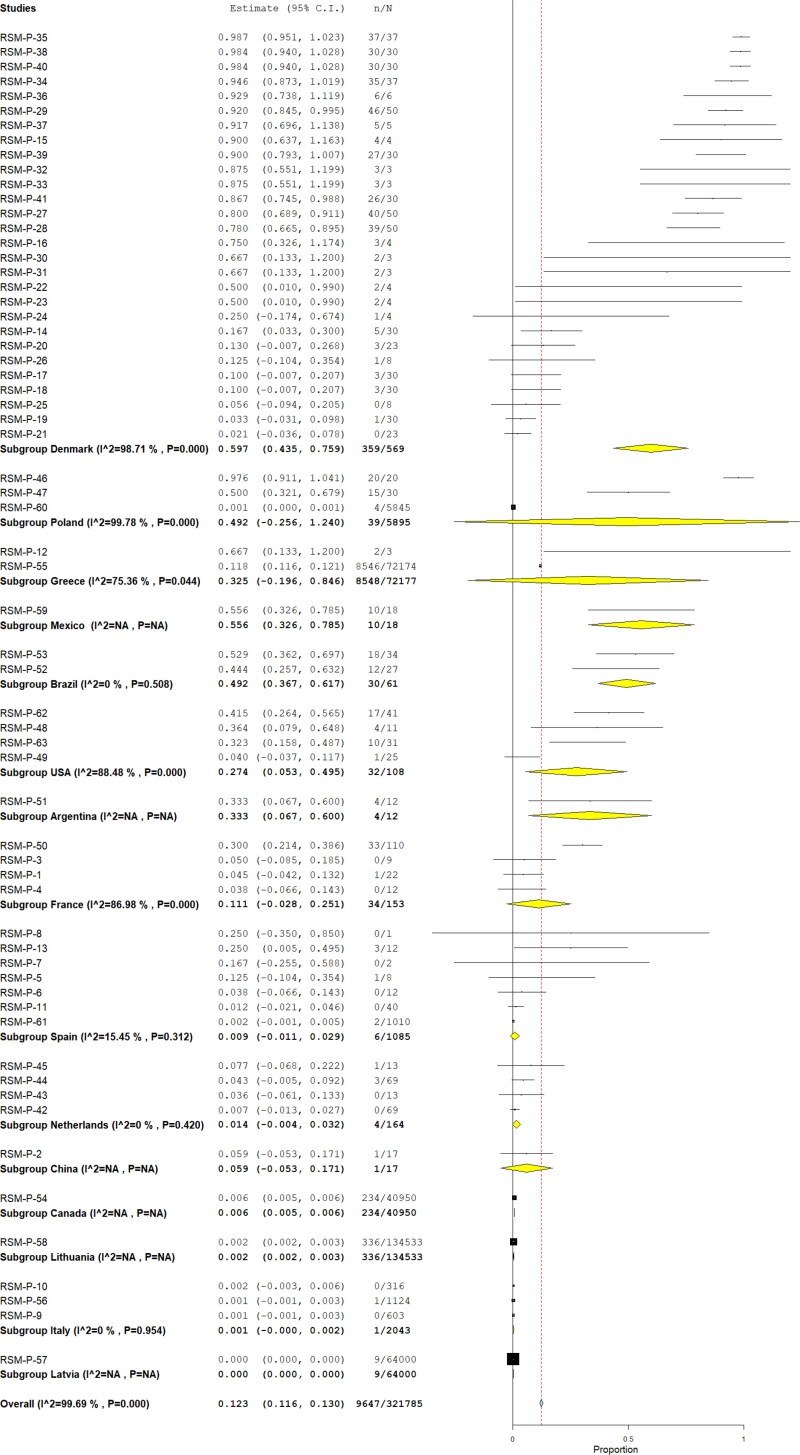
Pool prevalence of SARS-CoV-2 among animals assessed by RT-PCR from prevalence studies, by countries.

**Figure 4.**
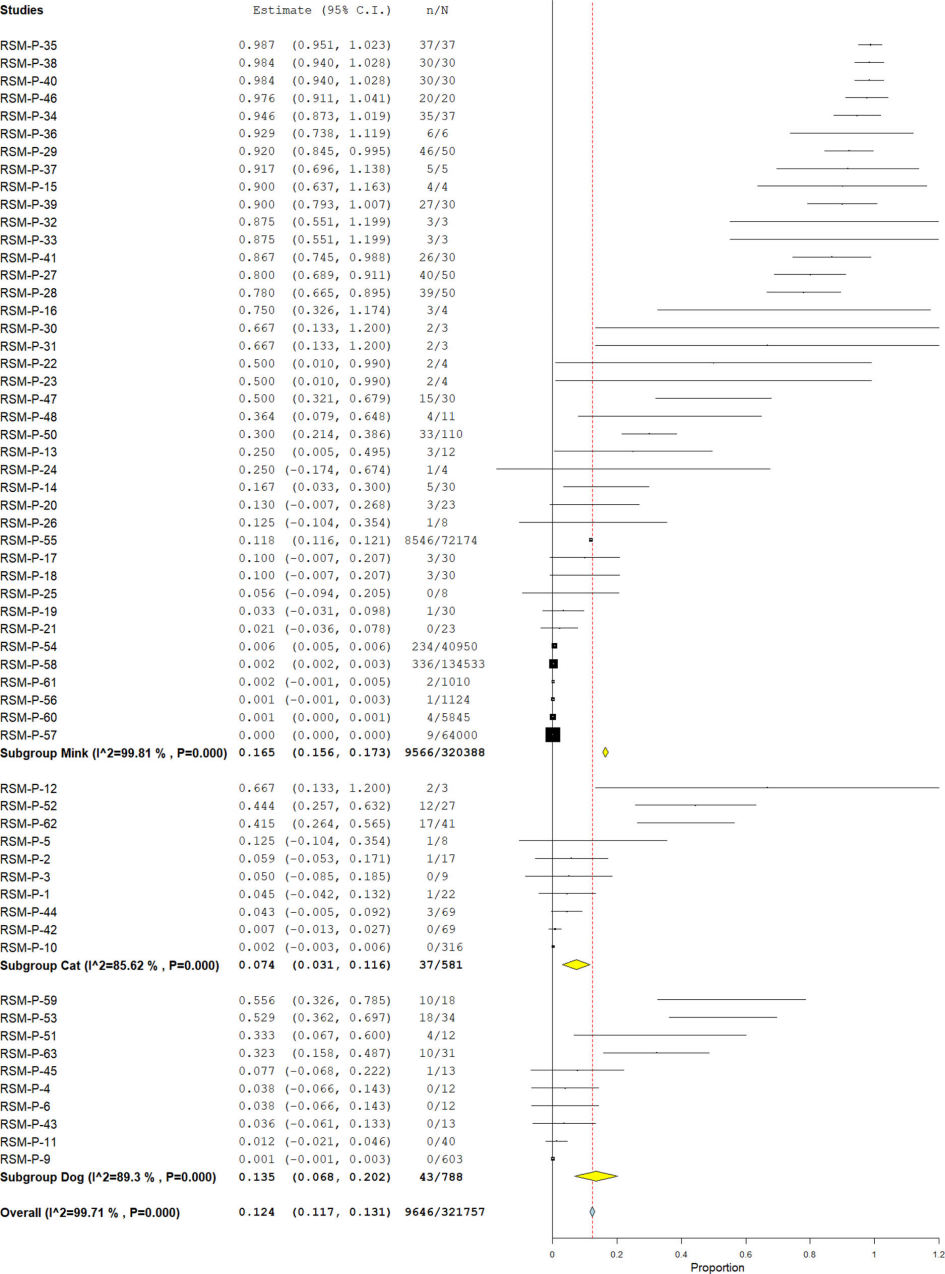
Pool prevalence of SARS-CoV-2 among animals assessed by RT-PCR from prevalence studies, by animal type.

**Figure 5.**
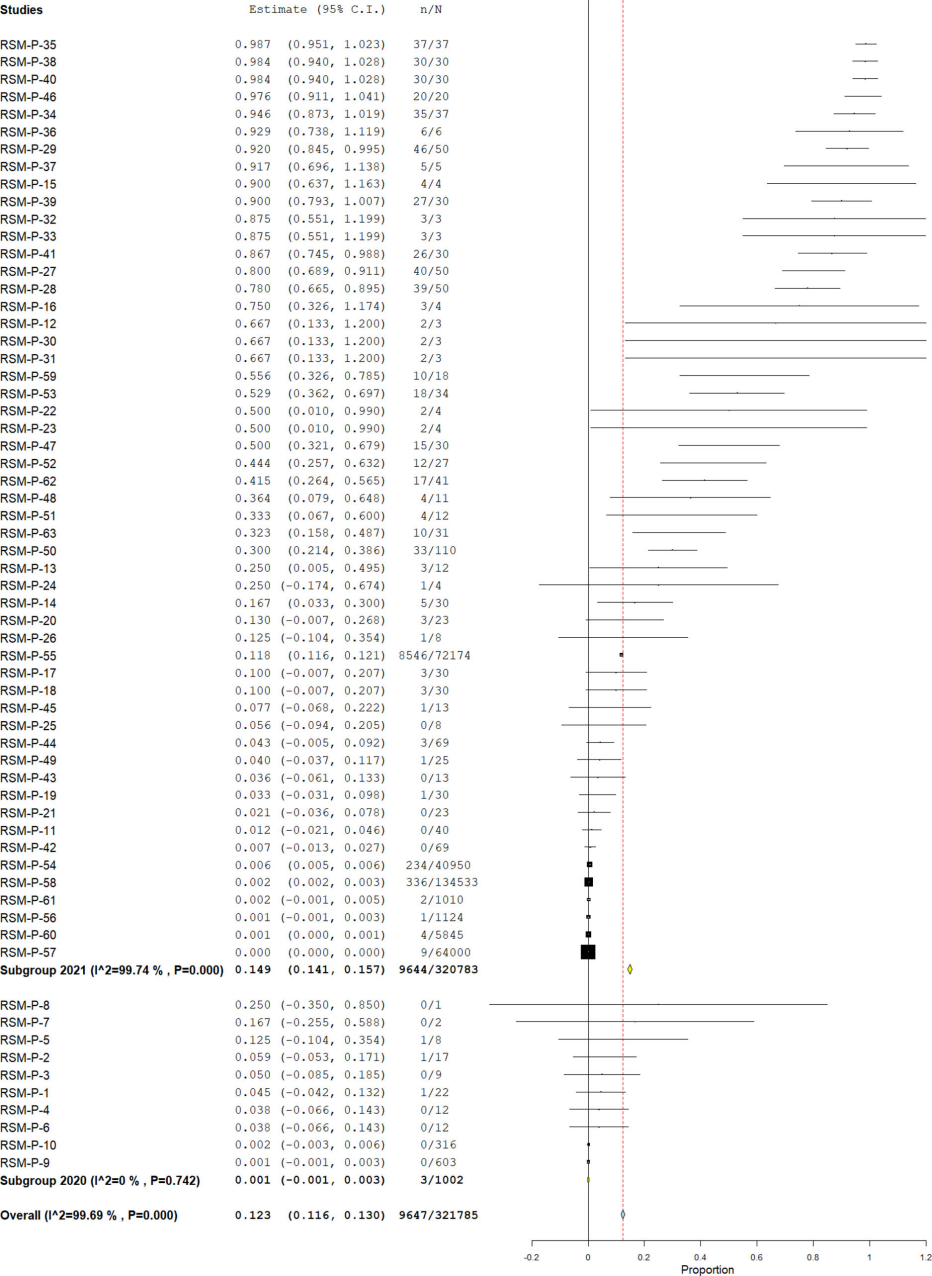
Pool prevalence of SARS-CoV-2 among animals assessed by RT-PCR from prevalence studies, by year.

**Figure 6.**
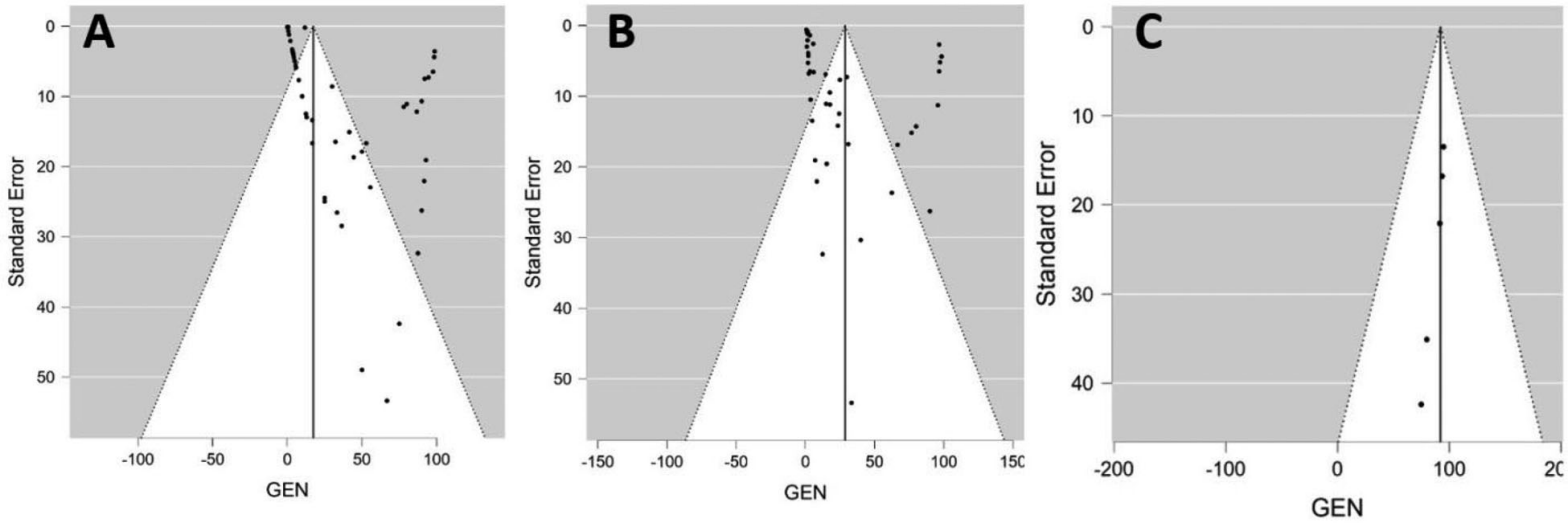
Funnel-plot for the Standard Error to assess for publication bias for the prevalence studies using RT-PCR (A), using serological tests (B), and for the case series (C).

Table 1 summarized the pool prevalences of SARS-CoV-2 among the case series, considering different variables such as origin of countries (Figure 3), type of animals (Figure 4), and year of study (Figure 5). Publication bias was assessed with a funnel plot for standard error, with suspicion of bias (Figure 6), both the Egger test (z = 16.707; *p* < 0.001), and Kendall's tau test (τ = 0.347; *p* < 0.001) indicated possible publication bias.

**Table 1.** Meta-analysis outcomes (random-effects model)*, for SARS-CoV-2 infection from case prevalence studies by RT-PCR and serological tests.

Technique	Variable	n	Pool prevalence (%)	95% CI	**Q** ^†^	**I^2^** ^‡^	τ**^2^**^§^	*p*
RT-PCR	All	321,785	12.3	11.6%-13.0%	20,168.30	99.693	<0.001	<0.001
	*Country*							
	Denmark	569	59.7	43.5-75.9	2090.098	98.71	–	<0.001
	Mexico	18	55.6	32.6-78.5	–	–	–	–
	Poland	5895	49.2	0.0-100.0	889.608	99.78	–	0.197
	Brazil	61	49.2	36.7-61.7	0.438	0	–	<0.001
	Argentina	12	33.3	6.7-60.0	–	–	–	–
	Greece	72,177	32.5	0.0-84.6	4.058	75.36	–	0.221
	USA	108	27.4	5.3-49.5	26.043	88.48	–	0.015
	France	153	11.1	0.0-25.1	23.05	86.98	–	0.117
	China	17	5.9	0.0-17.1	–	–	–	–
	Netherlands	164	1.4	0.0-3.2	2.822	0	–	0.120
	Spain	1085	0.9	0.0-2.9	7.097	15.45	–	0.385
	Canada	40,950	0.6	0.5-0.6	–	–	–	–
	Lithuania	134,533	0.2	0.2-0.3	–	–	–	–
	Italy	2043	0.1	0.0-0.2	0.094	0	–	0.167
	Latvia	64,000	0.0	0.0-0.0	–	–	–	–
	*Animal*							
	Mink	320,338	16.5	15.6-17.3	20017.888	99.81	–	<0.001
	Dog	788	13.5	6.8-20.2	84.112	89.3	–	<0.001
	Cat	581	7.4	3.1-11.6	62.598	85.62	–	<0.001
	*Year*							
	2020	1002	0.1	0.0-0.3	5981	0	–	0.293
	2021	320,783	14.9	14.1-15.7	20,162.137	99.74	–	<0.001
Serology	All	5319	29.4	22.9-35.9	11,203.039	99.607	0.044	<0.001
	*Country*							
	Denmark	354	62.8	34.3-91.2	2330.892	99.53	–	<0.001
	France	248	29.1	0.0-81.9	562.055	99.29	–	0.281
	Poland	270	27.6	22.3-33.0	0.844	0.00	–	<0.001
	China	1028	20.7	2.9-38.5	38.418	94.79	–	0.023
	USA	100	19.8	0.0-42.2	255.376	97.65	–	0.084
	Spain	167	7.1	0.0-20.1	5.446	81.64	–	0.280
	Netherlands	1255	4.6	1.9-7.2.0	34.552	79.74	–	<0.001
	Italy	919	4.3	2.0-6.5	2.537	60.58	–	<0.001
	*Animal*							
	Mink	815	62.6	40.4-84.7	3109.060	99.52	–	<0.001
	Cat	2072	8.5	5.5-11.4	92.222	89.16	–	<0.001
	Dog	2216	3.3	1.4-5.2	37.360	73.23	–	<0.001
	*Year*							
	2020	2028	5.2	2.4-8.1	51.495	84.46	–	<0.001
	2021	3291	35.2	25.4-44.9	10,588.176	99.67	–	<0.001

*95% CI = 95% confidence interval. † Cochran's Q statistic for heterogeneity. ‡ I^2^ index for the degree of heterogeneity. § Tau-squared measure of heterogeneity. *Some studies assessed simultaneous variables. Multiple studies assessed the prevalence by different methods.

### 3.3. Serological findings from prevalence studies

**Figure 7.**
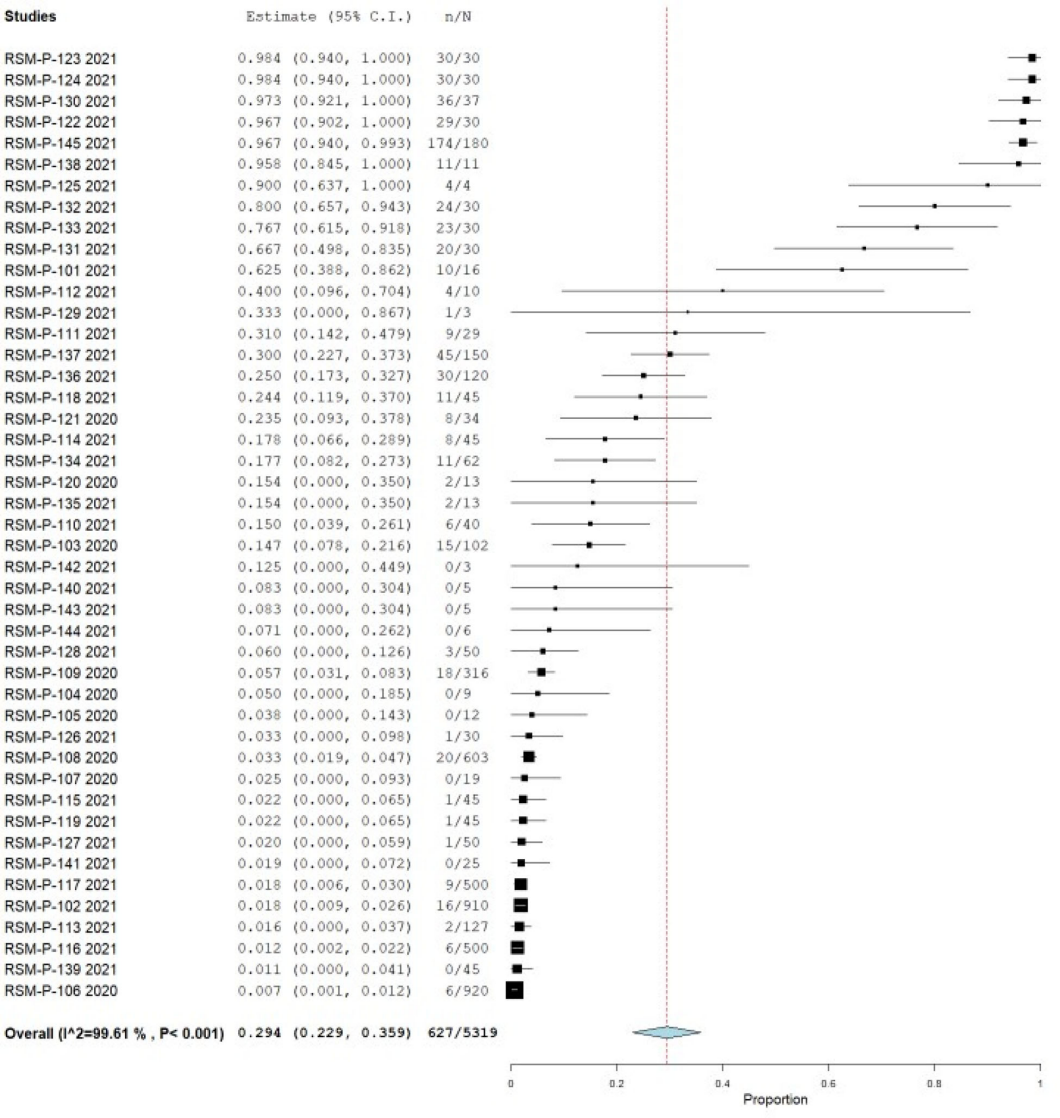
Pool prevalence of SARS-CoV-2 among animals assessed by serological tests from prevalence studies.

**Figure 8.**
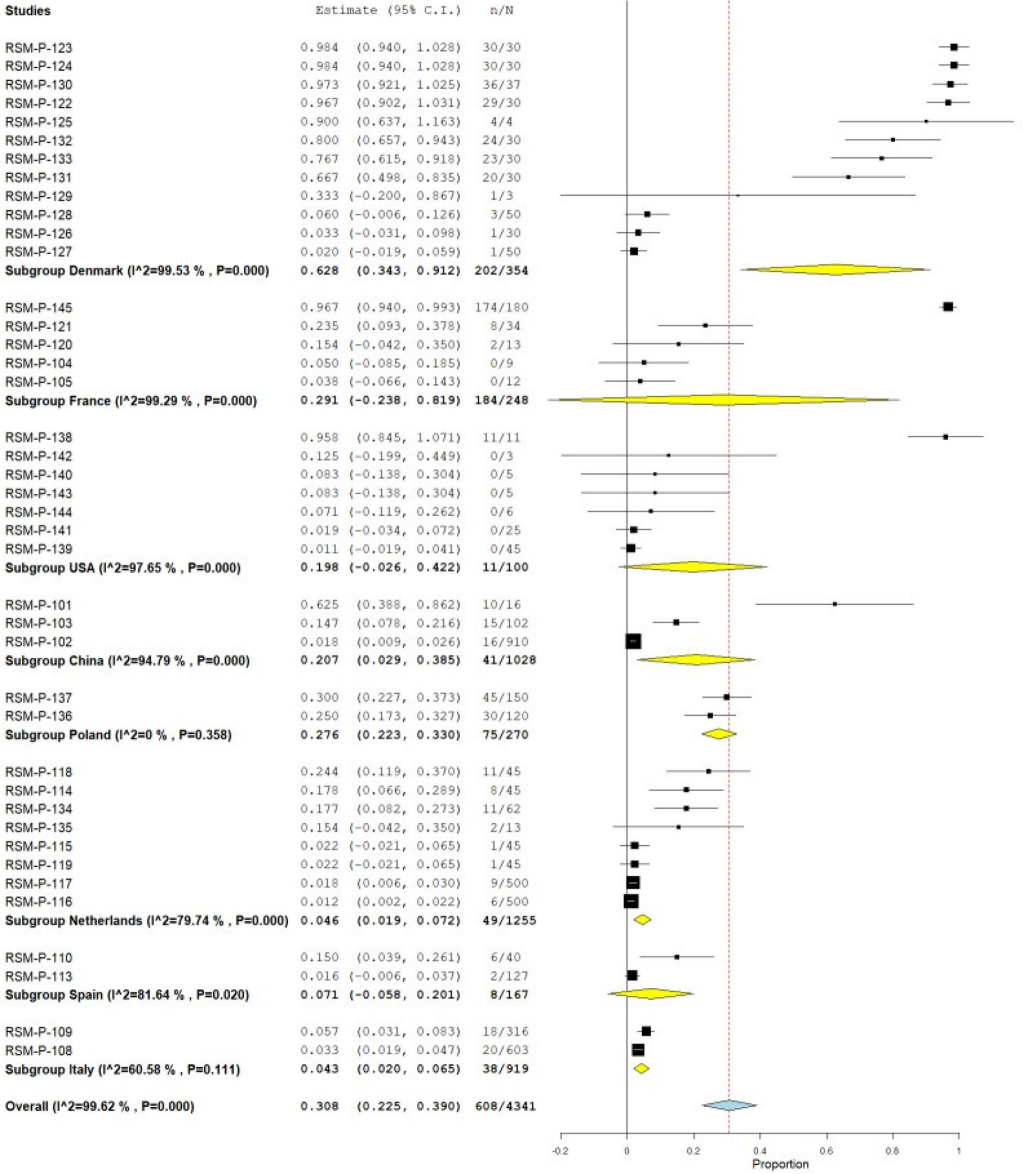
Pool prevalence of SARS-CoV-2 among animals assessed by serological tests from prevalence studies, by country.

**Figure 9.**
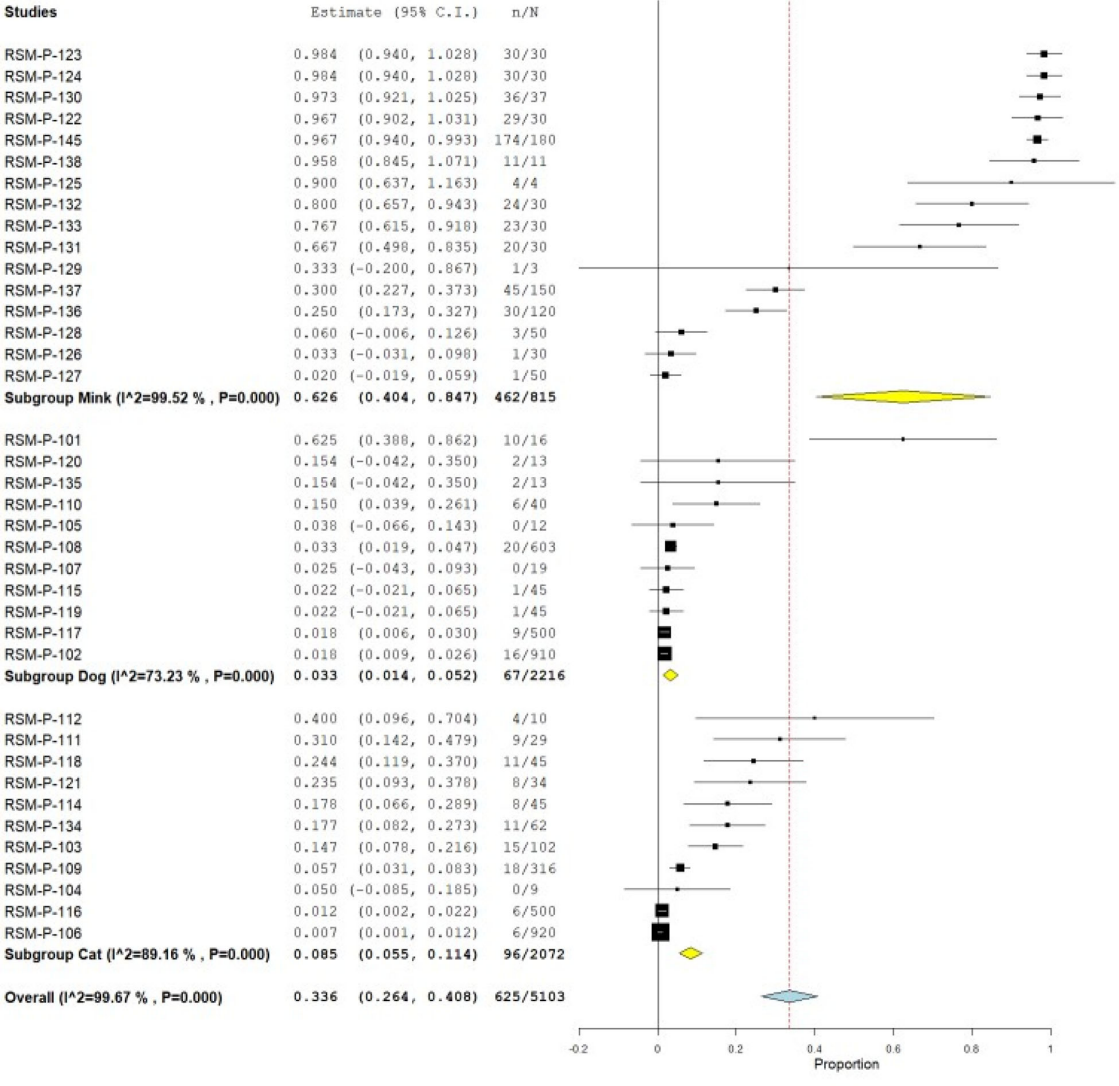
Pool prevalence of SARS-CoV-2 among animals assessed by serological tests from prevalence studies by animals.

**Figure 10.**
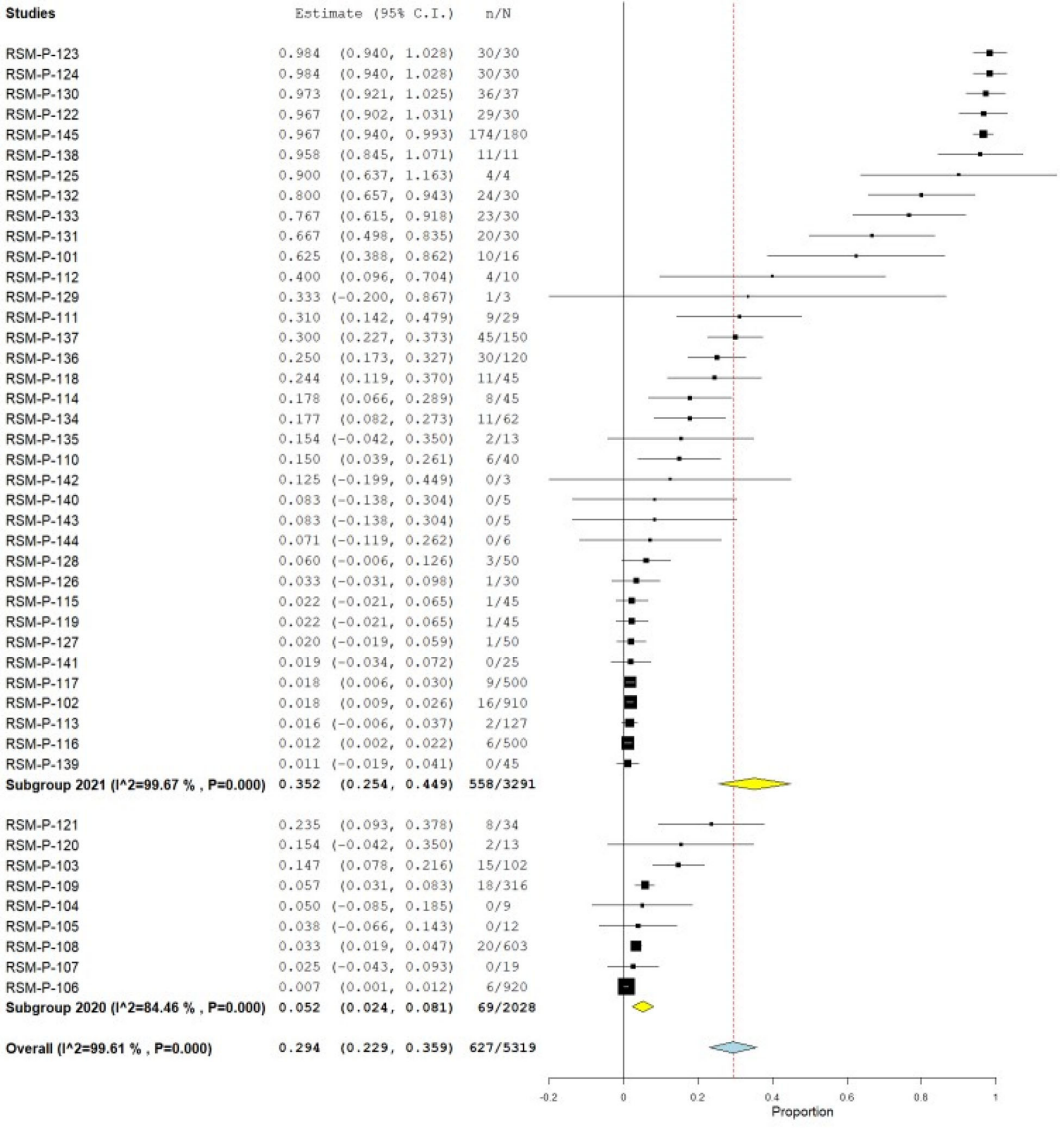
Pool prevalence of SARS-CoV-2 among animals assessed by serological tests from prevalence studies, by years.

Also, a total of 17 studies additionally assessed serological response against SARS-CoV-2, including nine by ELISA, four by Enzyme-Linked ImmunoSorbent Assay (ELISA), four by Plaque Reduction Neutralization Test (PRNT), one by Magnetic Immunoassay (MIA), one by immunochromatography (rest, two studies, the method was not specified), combining a total of 5319 animals, being 627 positives, yielding a pooled prevalence of 29.4% (95% CI 22.9%–35.9%) (Q = 11,203.039; I^2^=99.607; τ^2^=0.044; *p* < 0.001) (Figure 7). Four of these publications reported clinical findings, combining 71 animals from the 46 (65% had clinical findings). Regarding the origin, 83% were from pets/domestic animals, 15.3% farm animals, and 1.7% wild animals. Table 1 summarized the pool prevalences of SARS-CoV-2 among the case series, considering different variables such as origin countries (Figure 8), type of animals (Figure 9), and years of study (Figure 10). Publication bias was assessed with a funnel plot for standard error, with no suspicion of bias (Figure 6), the Egger test did not suggest possible publication bias (z = 1.579; *p* = 0.114), but Kendall's tau test did (τ = 0.307; *p* < 0.001).

### 3.4. Molecular findings from case series

**Figure 11.**
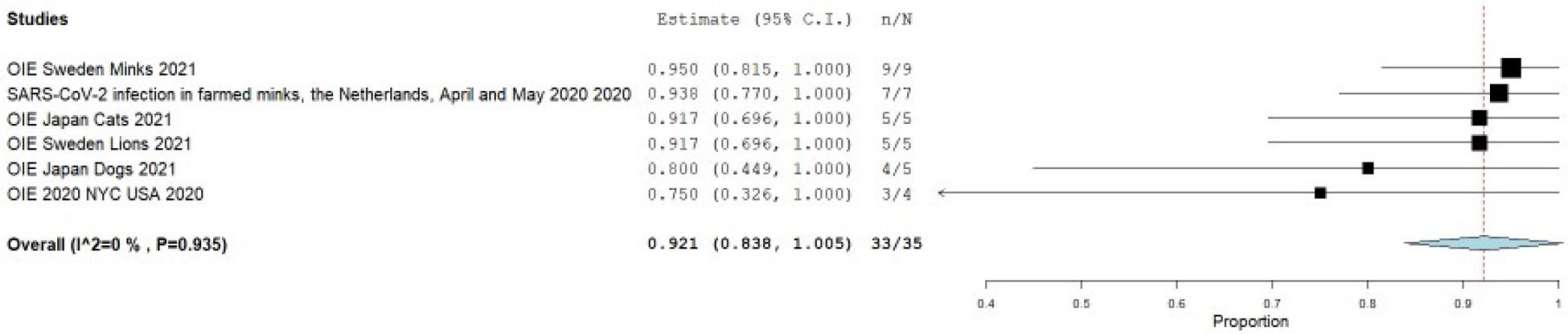
Pool prevalence of SARS-CoV-2 among animals assessed by RT-PCR at case series.

**Figure 12.**
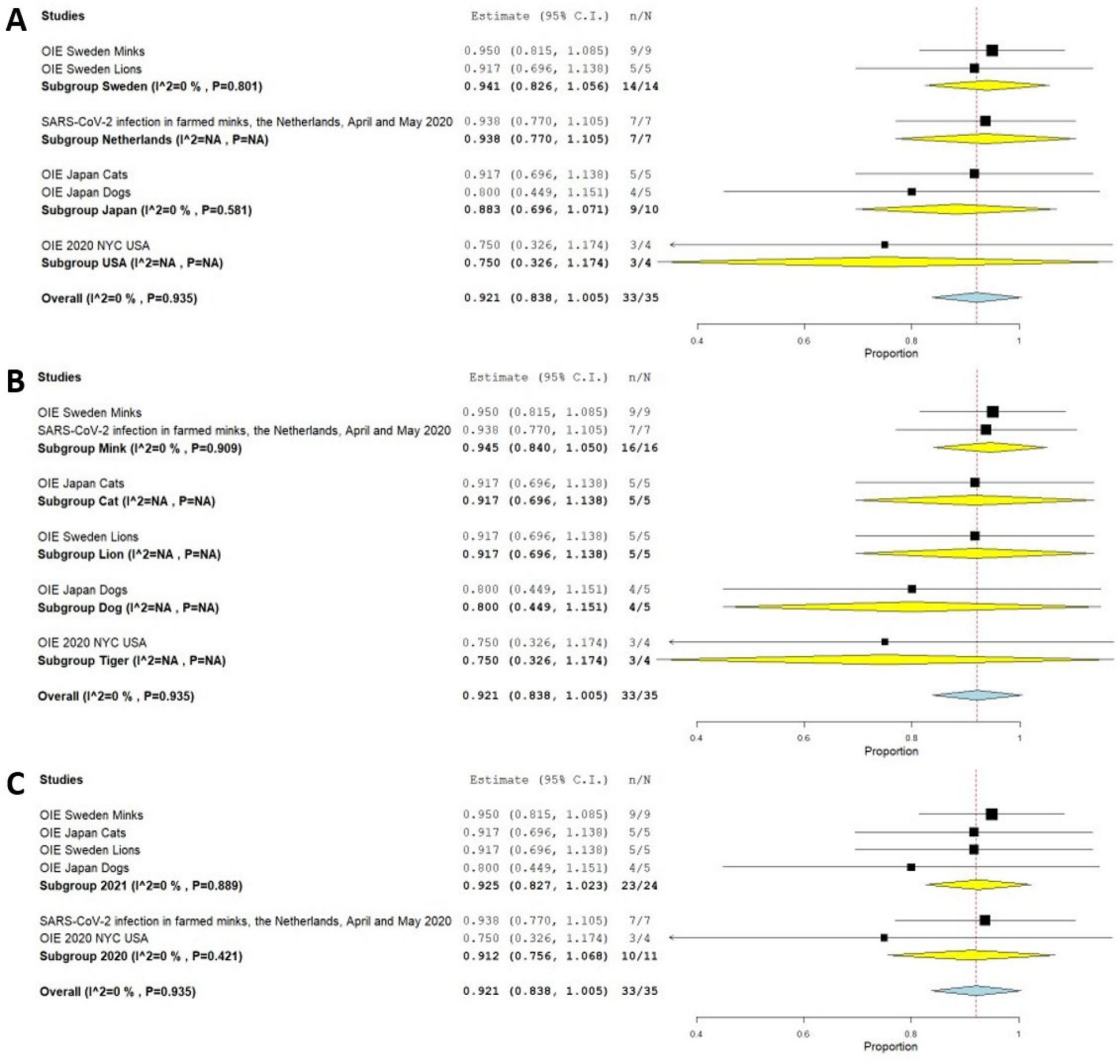
Pool prevalence of SARS-CoV-2 among animals assessed by RT-PCR at case series by countries (A), by animals (B), and by years (C).

We included a total of 6 case series that combined a total of 35 animals where RT-PCR investigated SARS-CoV-2 and 33 of them were positive, that yielded a pooled prevalence of 92.1% (95% CI 83.8%–100.0%) (Q = 1.298; I^2^=0.0; τ^2^<0.001; *p* < 0.001) (Figure 11); these were assessed by nasal swab sampling in 56% of them, 33% oral, and 11% rectal. From the positive animals (33), 24 (73%) showed clinical findings. Table 2 summarizes the pool prevalences of SARS-CoV-2 among the case series, considering different variables such as origin countries (Figure 12), type of animals (Figure 12), and years of study (Figure 12). Publication bias was assessed with a funnel plot for standard error, with no suspicion of bias (Figure 6), with an Egger test not suggesting possible publication bias (z=-0.567; *p* = 0.571), but Kendall's tau test was −1.000 (*p* = 0.006), indicating possible publication bias.

**Table 2.** Meta-analysis outcomes (random-effects model)*, for SARS-CoV-2 prevalence by RT-PCR, from case Series.

Variable	Number of Studies	Pool prevalence (%)	95% CI	n	**Q** ^†^	**I^2^** ^‡^	τ**^2^**^§^	*p*
All	6	92.1	83.8-100.0	35	1.298	0	<0.001	<0.001
*Countries*								
Sweden	2	94.1	82.6-100.0	14	0.064	0	–	0.801
Netherlands	1	93.8	77.0-100.0	7	–	–	–	–
Japan	2	88.3	69.6-100.0	10	0.304	0	–	0.581
USA	1	75.0	32.6-100.0	4	–	–	–	–
*Animals*								
Mink	2	94.5	84.0-100.0	16	0.013	0	–	<0.001
Cats	1	91.7	69.9-100.0	5	–	–	–	–
Lions	1	91.7	69.9-100.0	5	–	–	–	–
Dogs	1	80.0	44.9-100.0	5	–	–	–	–
Tigers	1	75.0	32.6-100.0	4	–	–	–	–
Year								
2020	2	91.2	75.6-100.0	11	0.649	0	–	<0.001
2021	4	92.5	82.7-100.0	24	0.631	0	–	<0.001

*95% CI = 95% confidence interval. † Cochran's Q statistic for heterogeneity. ‡ I^2^ index for the degree of heterogeneity. § Tau-squared measure of heterogeneity. *Some studies assessed simultaneous variables. Multiple studies assessed the prevalence by different methods.

### 3.5. Molecular and serological findings from case reports

We included a total of 50 case reports that combined a total of 78 animals where RT-PCR investigated SARS-CoV-2, and 64 of them were positive (82.05%) (76% of them by nasal swab sampling, 15% oral, 5% faecal, 2% vomit and 2% blood). Also, a total of 7 animals where serological tests assessed antibodies against SARS-CoV-2, including PRTN (1/1) and ELISA (1/3) (rest, 3/3, the method was no specified) resulting in 5/7 (71%) positive cases. Table 3 summarizes the characteristics of the SARS-CoV-2 RT-PCR positive animals (n = 64).

**Table 3.** Characteristics of the SARS-CoV-2 RT-PCR positive animals (n = 64) published as case reports.

Variable	N	%	Variable	N	%
*Country*			*Species*		
Canada	11	17.19	Cat	37	57.81
USA	9	14.06	Dog	10	15.63
Sweden	5	7.81	Tiger	5	7.81
Chile	4	6.25	Lion	4	6.25
China	4	6.25	Mink	4	6.25
Germany	4	6.25	Ferret	2	3.13
Switzerland	4	6.25	Puma	2	3.13
Argentina	3	4.69			
Croatia	3	4.69			
Belgium	2	3.13	*Scientific names*		
Italy	2	3.13	*Felis catus*	36	56.25
Slovenia	2	3.13	*Canis lupus familiaris*	11	17.19
UK	2	3.13	*Neovison vison*	4	6.25
Uruguay	2	3.13	*Panthera leo*	4	6.25
Bosnia	1	1.56	*Panthera tigris altaica*	2	3.13
Estonia	1	1.56	*Panthera tigris jacksoni*	2	3.13
France	1	1.56	*Puma concolor*	2	3.13
Russia	1	1.56	*Mustela furo*	1	1.56
South Africa	1	1.56	*Oryctolagus cuniculus*	1	1.56
Thailand	1	1.56	*Panthera tigris*	1	1.56
United Kingdom	1	1.56			
*Animal type*					
Pet	47	73.44	*Presentation with clinical findings*		
Wild	11	17.19	Symptomatic	38	59.38
Farm	6	9.38	Asymptomatic	8	12.50
Zoo	0	0.0	Not reported	18	28.13

Finally, using exclusively the database of the OIE, where reports of the animals which outcome was death, killed and disposed of, and slaughtered/killed for commercial use, are available, we proceeded to meta-analyze the proportions of assessed and infected animals that had those fatal outcomes. In 12 OIE reports, such data was available, combining 318,675 assessed animals with 9202 positives for SARS-CoV-2. One thousand two hundred ten were reported as deaths, and 17,776 were killed and disposed of; none were slaughtered/killed for commercial use. That yielded an overall population mortality (deaths/animal assessed) of 0.2% (95% CI 0.2%–0.3%) (Q = 1,197.999; I^2^=99.082; τ^2^<0.001; *p* < 0.001) (Table 4); an overall population sacrifice (killed/animal assessed) of 24.3% (95% CI 18.9%–29.7%) (Q = 70,136,993.187; I^2^=99.99; τ^2^=0.008; *p* < 0.001) (Table 4). Considering the infected animals, the overall case fatality rate (deaths/infected animals) yielded 1.4% (95% CI 1.1%–1.7%) (Q = 1,151.143; I^2^=99.131; τ^2^<0.001; *p* < 0.001) (Table 4); and finally, the overall proportion of killed infected animals (killed/infected animals) was of 26.4% (95% CI 0.0%–68.1%) (Q = 47,496,166.910; I^2^=99.99; τ^2^=0.491; *p* < 0.001) (Table 4).

**Table 4.** Meta-analysis (random-effects model)*, for SARS-CoV-2 related fatal outcomes of the assessed and infected animals.

Variable	n	Overall frequency (%)	95% CI	**Q** ^†^	**I^2^** ^‡^	τ**^2^**^§^	*p*
Mortality^a^	318,675	0.2	0.2-0.3	1198.00	99.082	<0.001	<0.001
Sacrifice^b^	318,675	24.3	18.9-29.7	70,136,993.19	99.99	0.008	<0.001
Case fatality rate^c^	9202	1.4	1.1-1.7	1151.14	99.131	<0.001	<0.001
Killed infected animals^d^	9202	26.4	0.0-68.1	47496166.91	99.99	0.491	<0.001

* 95% CI = 95% confidence interval.

^†^ Cochran's Q statistic for heterogeneity.

^‡^ I^2^ index for the degree of heterogeneity.

^§^ Tau-squared measure of heterogeneity.

^#^ Some studies assessed simultaneous variables. Multiple studies assessed the prevalence by different methods.

^a^ Mortality (deaths/animal assessed).

^b^ Sacrifice (killed/animal assessed).

^c^ Case fatality rate (deaths/infected animals).

^d^ Killed infected animals (killed/infected animals).

## 4. Discussion

The COVID-19 pandemic has affected over 214.5 million people globally, with more than 4.47 million deaths up to August 26, 2021 (Cimerman et al. 2020; Rodriguez-Morales, Katterine Bonilla-Aldana et al. 2020). Fortunately, since late 2000 some positive impact on the progress of this pandemic has been related to the deployment of anti-SARS-CoV-2 vaccines in different regions of the world, now with over 5.08 billion doses administered (Patel et al. 2020; Schlagenhauf et al. 2021).

Given the magnitude of such pandemic, in addition to the origins of the SARS-CoV-2, it is critical, considering the extent of human-animal contact, to understand the potential risk derived from the SARS-CoV-2 infected humans to animals (Bonilla-Aldana, Holguin-Rivera, et al. 2020; Halfmann et al. 2020). The main finding of the current meta-analysis indicated that around one in 8 animals suspected and assessed by RT-PCR for SARS-CoV-2 was positive. This is a remarkable proportion of active infection. Additionally, serological tests also found a high seroprevalence when the assessment was performed, indicating more exposure than infection and disease in almost a third of the animals. Also, in this context, serological cross-reactions may occur. Some authors suggest that considering the SARS-CoV-2 recombination rates (Haddad et al. 2021; Varabyou et al. 2021; Wang et al. 2021), the number of infected people and recent reports of environmental contamination (Hrudey et al. 2021; Mendes et al. 2021), the possibility of SARS CoV-2 transmission to animals can be expected more and more (Jemeršić et al. 2021). Indeed, in the current systematic review, in both molecular and serological analyses, the prevalence of SARS-CoV-2 was found higher in 2021 compared to 2020. This may be related to those factors and the progress of the pandemic and more studies approaching the actual situation of natural infection in animals from this emerging coronavirus.

From bats and pangolins, wild and non-wild animals have been on the radar of research-oriented efforts to describe the presence of SARS-CoV-2 infection, possible transmission and risk for humans (Brugère-Picoux and Shi 2021; Geldenhuys et al. 2021; Ma et al. 2021). However, early on in the pandemic, when cats and dogs appeared to be affected by this virus, the risk from human to domestic animals with first reports in Asia and Europe in 2020 and later (Sailleau et al. 2020; Zhang et al. 2020; Ruiz-Arrondo et al. 2021). In this meta-analysis, these domestic animals also had a considerable prevalence by molecular and serological tests, dogs (13.5% and 3.3%) and cats (7.4% and 8.5%). Nevertheless, as observed in the course of the pandemic in Europe, the impact seems to be higher among some farm animals, as it was the case of minks (*Neovison vison*), that generated multiple outbreaks and cluster infections in farms all over multiple countries in the continent leading in many cases to fatal outcomes (Enserink 2020; Molenaar et al. 2020). Interestingly, some early studies began to assess the clinical and pathological findings of those farmed minks that died from SARS-CoV-2 and found similar results that other authors detected in humans (Vasquez-Bonilla et al. 2020), e.g. diffuse alveolar damage with hyaline membranes (Molenaar et al. 2020; Vasquez-Bonilla et al. 2020). Indeed, the severity and frequency of SARS-CoV-2 infections in mink appear to be higher than in other animals. As observed from molecular and serological studies in this meta-analysis and case series, minks ranked first, with almost 1 out of 6 of the assessed by RT-PCR infected.

Additionally, with a seroprevalence above 62%, that may be low, as such studies corresponded fundamentally to findings during outbreaks, findings after outbreaks in non-culled animals and findings in at-risk farms with potentially missed outbreaks. Then, this may be is not necessarily representative for either the general commercially housed mink population or the wild population in any geographical area. Then, this requires more studies, as minks may become, after humans, the second most relevant susceptible hosts for SARS-CoV-2, and then, at the same time, a potential source for other animals, as has been shown by recent evidence in some studies about mink-to-cat transmission in the Netherlands (van Aart et al. 2021). In the end, also, with minks, as well as with dogs and cats, more studies regarding transmission to humans from these animals are required.

Multiple other animals have been reported to be infected, but the number and proportion seem to be considerably lower when compared to minks, dogs and cats. This is the case with other felines, that as expected, are also susceptible to SARS-CoV-2 infection (Mathavarajah and Dellaire 2020). In close contact with humans, lions and tigers at zoos were found in 2020 infected by SARS-CoV-2, especially in the USA (Bartlett et al. 2021; McAloose et al. 2020). In the case series and case report, these wild felines showed a high proportion of infection. However, the number of animals assessed is globally limited, probably leading to an overestimation of the infected proportion. In case reports, other felines, as is the case of the puma, are also reported (do Vale et al. 2021; Sharun, Dhama, et al. 2021; Sharun, Tiwari, et al. 2021). Also, in domestic environments, ferrets have been reported infected with SARS-CoV-2 (Giner et al. 2021).

In addition, a vast number of other animals may be added in similar systematic reviews in the future, as soon as more studies are available; as is the case for non-human primates (including gorillas), leopards, raccoon dogs, cynomolgus macaques, rhesus macaques, white-tailed deer, rabbits, Egyptian fruit bats, and Syrian hamsters, that are susceptible to infection with SARS-CoV-2 (Sharun, Dhama et al. 2021).

Considering the global epidemiology of COVID-19, some countries with a high incidence of the disease have still not even report a single case of SARS-CoV-2 infection in these animals. This is the case of India, Colombia, Turkey, Russia, among others that have reported more than 4 million human cases of COVID-19 during the pandemic. As a consequence of the animals assessed massively in some countries, the prevalence was higher in Denmark (Larsen et al. 2021). There is a lack of studies in Latin America, a region severely affected by the pandemic, except Mexico, Brazil, Argentina, and Chile (Calvet et al. 2021), and now Argentina where a recent study detected the SARS-CoV-2 infection in 18 cats and 20 dogs from owners previously confirmed as COVID-19-positive, including genome sequencing, B.1.499, a lineage reported in different provinces of the country (Fuentealba et al. 2021).

Finally, the clinical presentation and outcome of the animals are remarkable. At molecular and serological studies, more than 41% of the animals presented with clinical findings. A recent review focused on clinical outcomes also concluded, especially in felines, that the clinical signs they developed had a similar progression to those occurring in humans, suggesting a relationship between the viral cycle and target tissues of the virus in different species, which is true probably in certain species. Also, the cycles and target tissues seem to be comparable between different species (Giraldo-Ramirez et al. 2021). This is also consistent with the fact that among those confirmed cases by RT-PCR reported to the OIE; the case fatality rate was above 1%. Furthermore, the proportion of infected animals killed was more than 26% (mainly minks and other species). This is concerning, as mentioned before, suggesting that SARS-CoV-2 can infect these animals and produce clinical disease with fatal outcomes in a considerable proportion of these animals, including mortality (0.2%), sacrifice (24.3%), lethality (1.4%) and killing (26.4%) (Table 4). Given the risk in many cases, there is a mandatory culling of infected animals. As consequence of many mink outbreaks in Europe, these are culled. Also, the regulations have led to either an increase in detected cases, and in reported cases. Nevertheless, a limitation of such data, is these estimators were based mainly in data derived from OIE reports, as stated in the methods paragraph and Table 4. For reference, the case fatality rate in humans globally has been around 2–3%, with the highest values in countries with elderly populations and higher proportions of people with other risk factors (e.g. diabetes, hypertension) (Rodriguez-Morales, Cardona-Ospina, et al. 2020), also suggesting another field for future study among infected animals, such as dogs and cats, that frequently suffer also from these chronic diseases (Forrat et al. 1998). Thus, risk factors for severe and fatal COVID-19 in humans may be shared with animals? Nevertheless, in many outbreaks, even in the most sensitive animal species (minks), there is a lack of clinical disease in most animals, as is also reported in recent OIE reports from many countries.

Many questions can be raised from the current level of cumulated evidence regarding the SARS-CoV-2 natural infection in animals. However, with the data available, there is an urgent need to consider its potential importance in transmission, interspecies, from human-to-animals, One Health perspectives that integrate human and animal health, when assessing cases occurring in domestic, farm and zoos, environments, integrated surveillance and the need for increase regular testing among animals, beyond just research. There is a need to standardize molecular and serological tests for SARS-CoV-2 among animals (Lau et al. 2021), allowing these to be offered to the owners and increasing the diagnosis. At the same time, there is space for the discussion of more active surveillance, instead of a passive report to OIE from the countries, promoting the searching of animal cases among the cluster of human cases. SARS-CoV-2/COVID-19 deserve a comprehensive approach from the One Health approach (Leroy et al. 2020; Lorusso et al. 2020; Dasgupta et al. 2021). More integration is still needed to increase our understanding of transmission, risks and consequences of this emerging coronavirus disease. Finally, now that vaccination, also specifically on zoo animals in the USA, have started (over 11,000 doses at 70 zoos from 27 states), it would be interesting to see its impact in the near future in effectiveness and protection SARS-CoV-2 infection (Reuters 2021).

## 5. Limitations

In this study, we did not differentiate clearly studies assessing the prevalence in screenings and the prevalence in outbreaks in closed groups of animals. Some studies were performed in the context of outbreaks but were not specified. Nevertheless, then, the data for it is still limited. Subsequently, additional analysis should also be performed in the future, with more available and specific studies. In future assessments, it would be good to have a clear distinction between studies testing randomly in the wild population where animals are tested or when found dead and animals in commercial situations (tested when there appears to be an outbreak) or companion animals (tested often concerning positive RT-PCR results of their owners) all of which has a high impact on the chances of finding a RT-PCR-positive result. Doubtless, a more in-depth analysis of this would be interesting. Finally, there is a need for a thorough review of SARS-CoV-2 natural infections in animal species, emphasizing how to interpret the findings of other authors in future systematic reviews and meta-analyses.

## Author contributions

AJRM and DKBA formulated the research questions, designed the study, developed the preliminary search strategy, and drafted the manuscript. AGB, SDJD, JBL, MCCT, LASM refined the search strategy by conducting iterative database queries and incorporating new search terms. AGB, SDJD, JBL, MCCT, LASM, AJRM, and DKBA searched and collected the articles. AJRM and DKBA conducted the quality assessment. All authors critically reviewed the manuscript for relevant intellectual content. All authors have read and approved the final version of the manuscript.

## Ethical approval

Approval was not required.

## Data Availability

Data is available upon a reasonable request.
